# Mutations at positions 186 and 194 in the HA gene of the 2009 H1N1 pandemic influenza virus improve replication in cell culture and eggs

**DOI:** 10.1186/1743-422X-7-157

**Published:** 2010-07-14

**Authors:** Pirada Suphaphiphat, Michael Franti, Armin Hekele, Anders Lilja, Terika Spencer, Ethan Settembre, Gene Palmer, Stefania Crotta, Annunziata B Tuccino, Bjoern Keiner, Heidi Trusheim, Kara Balabanis, Melissa Sackal, Mithra Rothfeder, Christian W Mandl, Philip R Dormitzer, Peter W Mason

**Affiliations:** 1 Novartis Vaccines and Diagnostics, Cambridge, MA, USA, Siena, Italy, and Marburg, Germany; 2 National Institute of Medical Research, The Ridgeway, NW7 1AA, London, UK

## Abstract

Obtaining suitable seed viruses for influenza vaccines poses a challenge for public health authorities and manufacturers. We used reverse genetics to generate vaccine seed-compatible viruses from the 2009 pandemic swine-origin influenza virus. Comparison of viruses recovered with variations in residues 186 and 194 (based on the H3 numbering system) of the viral hemagglutinin showed that these viruses differed with respect to their ability to grow in eggs and cultured cells. Thus, we have demonstrated that molecular cloning of members of a quasispecies can help in selection of seed viruses for vaccine manufacture.

## Findings

In the spring of 2009, a novel type A influenza virus of swine origin (S-OIV) bearing an H1 hemagglutinin (HA) and an N1 neuraminidase (NA) was isolated from acutely ill humans [1]. Unlike some novel influenza strains, such as recent H5N1 strains of avian origin, S-OIV is not highly pathogenic [2]. However, it is readily transmissible and has spread globally in a new pandemic. Young people and those with medical conditions, such as asthma, are at particularly high risk for morbidity and mortality from S-OIV [3]. The public health response to the emergence of S-OIV has been swift, with the rapid manufacture, testing, and distribution of monovalent pandemic vaccines and inclusion of the pandemic strain in the trivalent vaccine composition recommended for use in the 2010 southern hemisphere seasonal immunization campaign.

One of the great challenges to influenza vaccine manufacture is the rapid generation of safe and well growing seed viruses that are antigenically matched with newly emerged strains. Currently, selection of seasonal type A vaccine strains relies on a network of laboratories that generate classical reassortant viruses in eggs. To make these reassortants, the protective HA and NA determinants are swapped through mating onto the genetic background of a virus that grows to high titers in eggs. This method creates significant genetic bottlenecks and can produce variants with egg-adapted HA mutations that alter antigenic properties, making them unacceptable for use as vaccine seeds (see more below). The selection of variants under these circumstances undoubtedly relates to the quasispecies nature of the virus used to derive these reassortants.

Reassortants can also be generated by reverse genetics technology, in which viable infectious virus is rescued from cells transfected with plasmid DNAs encoding the 8 influenza virus genome segments [4,5]. For example, reverse genetics has been used in human vaccine manufacture to generate seeds for vaccines against highly pathogenic H5N1 avian-origin influenza viruses. Since these wild-type, high-pathogenicity avian strains kill the chick embryos used for manufacture, an engineered modification to the HA of these strains was used to lower their pathogenicity, permitting vaccine production in eggs [6].

Here, we describe the rescue of reverse genetics reassortants that carry the NA gene and one of three different HA genes derived from the A/California/04/2009 (termed A/CA/04/2009) isolate of S-OIV on a standard vaccine-compatible genetic background (A/PR/8/34). The three HA variants are members of the quasispecies represented in the RNA prepared from this A/CA/04/2009 clinical isolate that had been passed once in Madin Darby canine kidney (MDCK) cells. The different growth properties of the three rescued reassortants indicate that a reverse genetics seed development process that takes advantage of the natural diversity represented in influenza virus quasispecies may have advantages over methods (such as classical reassortant methods) that examine clonally derived virus strains for their utility as high yielding seeds for vaccine manufacture.

Our reverse genetics system is based on the dual-promoter plasmid system in which the A/PR/8/34 PB1, PB2, PA, NP, M and NS genes were cloned into the pHW2000 plasmid (or a plasmid with similar genetic elements) using universal primers [7]. In this system, the resulting plasmids contain influenza virus sequences that are transcribed from a cytomegalovirus (CMV) immediate early promoter to make mRNAs for protein expression and from a short version of the human RNA polI promoter to make negative-sense RNAs that can be replicated by the viral RNA-dependent RNA polymerase to form influenza virus genome segments [8]. Preliminary studies demonstrated that our plasmid system could generate reverse genetics influenza viruses in both human cells (293T) and in a derivative of MDCK cells that had been adapted to growth in suspension for vaccine manufacture [9]. Viral RNA (prepared from virus passaged twice in MDCK cells by using the QIAamp Viral RNA Mini Kit) was converted to cDNA using the Monsterscript reverse transcriptase (Epicentre Biotechnologies) and then amplified by overlap PCR using the Herculase polymerase (Stratagene) to create genomic segments corresponding to the posted sequence of this isolate [GenBank: GQ117044] and restriction sites compatible with the pHW2000 plasmid [7] (Oligonucleotide sequences are available from the authors upon request). *BsmBI*-digested PCR fragments were ligated to an appropriately digested pHW2000 vector DNA and transformed into E.coli. Ampicillin-resistant colonies were selected and screened by PCR and used without further analysis or re-streaking.

**Figure 1:**
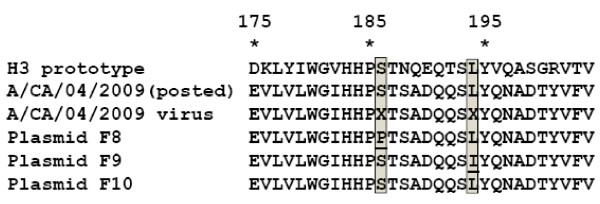
**Alignment of amino acid sequences of the S-OIV H1 HA with the H3 prototype HA in the region near the sialic acid binding site**. Protein sequences translated from the deposited S-OIV A/CA/04/2009 sequence [GenBank: GQ117044] are shown under the prototype of the H3 subtype from human (A/HongKong/1/68) strain sequence [PDB: 1HGD]. Below these are the sequences detected in cDNA amplified from A/CA/04/2009 RNA, with the prominent quasispecies at positions 186 and 194 indicated by "X," and three plasmid DNAs cloned from this quasispecies. Underlining indicates variations from the posted sequence.

Sequencing of RT-PCR amplified cDNA from A/CA/04/2009 (a strain of S-OIV) revealed a quasispecies in the HA gene, with clearly detectable heterogeneity in codons 186 and 194. Reassortant viruses were rescued by transfecting 293T or MDCK cells with plasmids encoding S-OIV HA and NA, the remaining six genome segments from A/PR/8/34, and the gene for TMPRSS2 serine protease [10] to help amplify rescued viruses. The NA used for the rescue differs from the posted A/CA/04/2009 NA sequence [GenBank: FJ969517] by a single mutation, which results in a N2S amino acid substitution. This mutation represents a natural existing variant of the virus that was amplified by chance. The three different A/CA/04/2009 HA-encoding plasmids used in the transfections represented different clones of the A/CA/04/2009 HA quasispecies: plasmid F8 encodes P186 and L194; plasmid F9 encodes S186 and I194; and plasmid F10 encodes S186 and L194, which matches the posted sequence [GenBank: GQ117044] (Fig. 1). These positions are based on the H3 numbering system.

Transfection of either 293T or MDCK cells with plasmid cocktails containing any of the HA variants produced reassortant viruses. Recovery in 293T cells was more efficient than in MDCK cells, with higher titers detectable in the original transfection supernatant. However, with modified recovery methods [9], all transfections produced virus from each of the HA plasmid DNAs. No infectious virus was recovered from any simultaneous control transfections with plasmid mixtures lacking a HA gene. Sequence analyses of HA and NA genes of the viruses rescued from the plasmid DNAs revealed that they faithfully retained sequences of the parental plasmid DNA (see Fig. 1).

**Figure 2:**
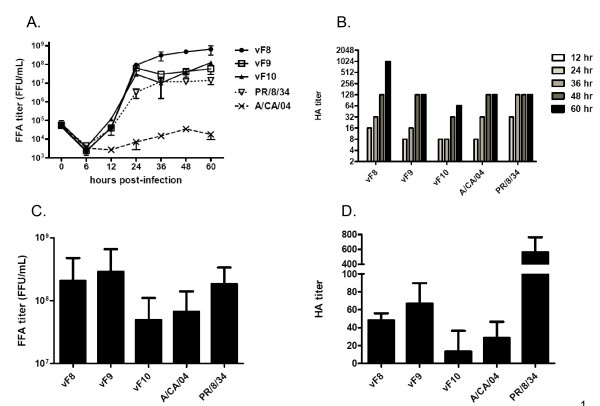
**Yield of A/CA/04/2009, A/PR/8/34, and reverse genetics viruses bearing the A/CA/04/2009 NA and selected HA genes, as described in Fig. 1**. **A. **Infectious titer of virus produced by MDCK cell monolayers infected at an MOI of 0.05 in 6-well plates, as determined by focus formation assay (FFA) on MDCK cells [21]. Cells were incubated at 37°C for the indicated times, after which 200 μl of the culture medium was sampled and stored at 4°C for infectious titer determination on MDCK cells using a slight modification of previously described FFA methods [21]. Data shown are the mean and standard deviation (S.D.) from one of three representative experiments. **B. **HA titers of MDCK-derived virus samples collected from the experiment in panel A, determined using standard methods [22]. Data shown are from one of three representative experiments. **C. **Infectious titers of virus produced by twelve- to fourteen-day-old embryonated chicken eggs infected with 100 μl of the indicated virus stocks containing 10,000 infectious units (as determined by FFA). The eggs were incubated for 72 hrs at 34°C after infection, and titers were determined as described for panel A. Mean and S.D. from four independent experiments are shown, with 2-4 eggs used for each virus in each experiment. **D. **HA titers of egg-derived virus samples collected from the experiment in panel C. Mean and S.D. from four independent experiments are shown.

Growth of the three reassortants (vF8, vF9, and vF10) was compared to the growth of wild type S-OIV and A/PR/8/34 in MDCK cells (Fig 2a and 2b) and embryonated chicken eggs (Fig 2c and 2d). Virus titer was assayed by formation of infectious foci on MDCK cells (focus formation assay - FFA) and guinea pig red blood cell agglutination (hemagglutination assay - HA). The wild-type S-OIV used for these studies was precisely the same virus used to produce the cloned plasmid DNAs, and it had been passaged only twice in MDCK cells. Interestingly, it produced approximately 10,000-fold less infectious virus on MDCK cells than it did in eggs (Fig. 2a and 2c), although it produced similar HA activity in eggs and MDCK cells (Fig. 2b and 2d). The A/PR/8/34 strain used in these studies was derived from an egg-passaged isolate that had been extensively passaged in MDCK cells and grew to approximately 10-fold lower infectious titer in MDCK cells than in eggs (Fig. 2a and 2c)

The three reverse genetics reassortants rescued with different HA variants had reproducibly different growth characteristics when propagated in MDCK cells and eggs. The F10 variant was significantly less productive than F8 and F9 by both infectious and HA assays in MDCK cells and in eggs (Fig 2a-d). The F8 variant grew to approximately 10-fold higher infectious titer and produced more than 4-fold greater HA activity than the other reverse genetics reassortants in MDCK cells (Fig. 2a and 2b), although its performance was comparable to that of the F9 variant in eggs (Figs. 2c and 2d). These data indicate that the quasispecies variation in HA affects growth characteristics relevant for vaccine seed suitability.

To determine if the HA mutations at positions 186 and 194 altered the antigenicity of HA, this panel of viruses was subjected to the hemagglutination inhibition assay (HAI) using ferret antisera against A/CA/04/2009, A/CA/07/2009, or RG-15 (a reverse genetics-derived A/TX/05/2009-like strain). Deposited sequences for A/CA/04, A/CA/07, and RG-15 HA [GenBank: GQ117044, FJ969540, and GQ457487, respectively) give identical amino acid sequences from residues 87 to 199 (H3 numbering). The HAI titers of all of these antisera with each of our reverse genetics variants were greater than or equivalent to those obtained with A/CA/04/2009, whereas reaction of these variants with normal ferret sera or reaction of A/PR/8/34 virus with these test sera were undetectable (Table 1). Thus, all of our reverse genetics viruses were antigenically similar to the parental A/CA/04/2009 and A/CA/07/2009 viruses despite the presence of point mutations that improved growth. However, a reverse genetics virus equivalent to F8 with an additional N159 D mutation, which increases growth in eggs but decreases HAI titer, had 8-fold lower HAI titer than A/CA/04 (data not shown); thus, demonstrating that some mutations in S-OIV HA can simultaneously alter growth and antigenic structure, confounding vaccine manufacture.

**Table 1:** Effect of variations at residues 186 and 194 of A/CA/04/2009 HAs on reactivity with post-infection ferret sera

**HAI titer with selected ferret sera**^**1**^				
**Virus designation**	**RG15**	**A/CA/04**	**A/CA/07**	**Normal**

				
vF8	320, 640^2^	1280, 2560	1280, 2560	<40, <40
vF9	160, 320	640, 1280	640, 1280	<40, <40
vF10	320, 320	5120, 2560	5120, 2560	<40, <40
A/CA/04/2009	160, 160	640, 640	640, 640	<40, <40
A/PR/8/34	<40, <40	<40, <40	<40, <40	<40, <40

^1 ^H1N1 immune ferret sera were obtained from CDC. Ferrets were infected with RG15 (IDCDC RG15, a reverse genetics virus containing the A/TX/05/2009 HA [A/TX/05/2009 × A/PR/8/34], A/CA/04 (A/CA/04/2009, Lot # 2009-082, CDC#2009712049), or A/CA/07 (A/CA/07/2009, Lot #2009-089, CDC#2009712112). Normal (un-infected ferret) sera was obtained from Alpha Diagnostics Intl., Inc.

^2 ^Data shown are from two experiments in which all four sera were checked in parallel with all five antigens.

HAI tests were performed by standard methods using guinea pig red blood cells, receptor-destroying enzyme-treated serum, and 4 HA units of antigen [25].

Examination of the electropherograms prepared from sequencing reactions created with the RNA recovered from wild type (non-reassortant) S-OIV virus harvested at late time points in MDCK growth from a similar experiment failed to reveal a detectable change in the proportion of the various HA genotypes within the quasispecies. The stability of HA quasispecies distribution in the wild type virus despite significant variation in the growth of reassortants prepared from individual HA clones selected from the quasispecies suggests that molecular cloning of individual variants can accelerate adaptation to growth in new conditions. In this study, we have demonstrated the utility of cloning members of the quasispecies in generating a potential vaccine seed derived by methods compatible with manufacture in vaccine-approved MDCK cells.

**Figure 3:**
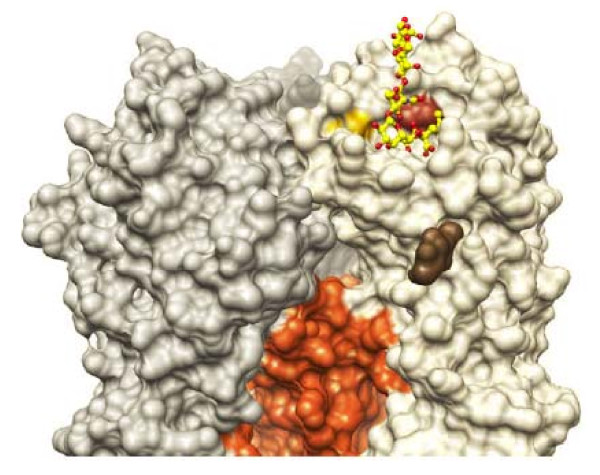
**Surface representation of A/CA/04/2009 HA modeled on the known structure of 1918 HA [PDB: **1RUZ**] with a sialoside bound at the active site (based on alignment to PDB: **1RVT**) **[11]. HA1 is shown in various shades of gray, HA2 in orange, and glycan modification of HA in brown. The bound sialoside is modeled as a ball-and-stick representation with yellow carbons and red oxygens. The positions of Ser186 (yellow) and Leu194 (red) are highlighted. The model was generated and the structure aligned with O [23], and the figure was generated with Chimera [24].

The variable 186 and 194 residues are immediately adjacent to the sialic acid binding site, based on a model we created by modeling the sequence A/CA/04/2009 HA onto the structure determined for the H1 HA from a 1918 pandemic strain of influenza virus [11] (Fig. 3). Therefore, these residues could affect cell attachment, substrate specificity, growth characteristics, and red blood cell agglutination. Similar mutations in H1N1 swine flu isolates from the 1970 s increase growth in eggs and MDCK cells and reduce growth in swine [12]. Other groups have also demonstrated positive selective pressure on a variety of residues near the receptor binding site in H3N2 isolates, including variations at the same residues (186 and 194) [13-15] that we detected in our H1 S-OIV quasi-species. More recently, Glaser et al. showed that a mutation at a nearby position (190) in the HA of the avian H1N1 virus that caused the 1918 pandemic was associated with this strain's adaptation to humans [16].

The HA sequence variants we detected in the A/CA/04/2009 isolate were not reported in two recent studies that examined variation in residues near the receptor binding pocket of many S-OIV isolates [17,18]. However, during the time this manuscript was being prepared, Chen et al. identified some natural HA variants (including L194I, but not S186P) in A/CA/04/2009 that improved virus recovery in MDCK/293 cells [19]. Carbohydrate microarray data do demonstrate differences in sialic acid specificity between S-OIV isolates that differ at several HA residues [20], suggesting that genetic pressure could be exerted on the receptor binding pocket of S-OIV during isolation on MDCK cells. Our data provide additional documentation of the importance of HA residues near the receptor binding pocket in the adaptation of viruses to growth *in vitro*. Because some of the variants increase the growth of S-OIV reassortants in mammalian cells and eggs, these results demonstrate that sampling viral quasispecies during the rescue of reassortant viruses by reverse genetics can identify useful isolates for vaccine manufacture.

## Competing interests

All authors are employees and/or shareholders in Novartis Vaccines and Diagnostics, which manufactures influenza vaccines in MDCK 33016-PF cells.

## Authors' contributions

PS carried out the rescue and characterization of virus reassortants, and helped draft the manuscript. AH, AL, BK, KB, MR, and ABT participated in the cloning and sequencing of viral genes. TS and MS participated in characterizing growth and antigenicity of virus reassortants. ES carried out the molecular modeling. MF, HT, CWM, PRD, and GP participated in the design and coordination of the study. PWM conceived the study and drafted the manuscript. All authors read and approved the final manuscript.
